# From sequencing to understanding: a grand challenge in genome-scale molecular and genetic analysis

**DOI:** 10.3389/fpls.2026.1782107

**Published:** 2026-02-06

**Authors:** Ruslan Kalendar

**Affiliations:** 1 Helsinki Institute of Life Science (HiLIFE), University of Helsinki, Helsinki, Finland; 2 Center for Life Sciences, National Laboratory Astana, Nazarbayev University, Astana, Kazakhstan

**Keywords:** evolution, genetical analysis, genome, high-molecular-weight DNA, long-read sequencing

## 1 The genome as an evolutionary system: repetitive elements, viruses, and the hidden genetic phenotype

The ultimate goal of genetic analysis is to determine the complete nucleotide composition and its variants in the genome under study in comparison with other individuals. Although most of the identified genetic variants are not associated with the phenotype, these data have other values related to the individual history of a particular genome. Multiple changes in the genomes of eukaryotes and prokaryotes carry the “historical experience” of interactions between viral infections and intragenomic mobile elements. There may be no differences in genes between individuals; however, the patterns of variants of various repetitive elements of viral or other origins may distinguish these individuals and reflect their unique “hidden” phenotype defined here as the cumulative signature of repetitive element insertions, deletions, and modifications that do not directly alter protein-coding sequences but may influence genome regulation, chromatin structure, and evolutionary potential (Palazzo and Gregory, 2014). These changes may reflect adaptive processes and evolutionary mechanisms that occur during the life of an organism. Analysis of such variations allows us to identify patterns related to disease resistance and responses to external factors (Arvas et al., 2023; Kalendar and Kairov, 2024; Kalendar et al., 2011; English et al., 2025; Kalendar et al., 2022). Thus, genetic analysis of all elements in the genome and their quantitative analysis serves not only to study hereditary traits, but also to understand the evolutionary history and biological function of the genome (Belyayev et al., 2010; Baumel et al., 2002; Kalendar et al., 2008, 2004; Lin et al., 2024). Plant genomes provide compelling examples of how repetitive elements shape genome evolution and adaptation. In wild diploid wheat (*Triticum dicoccoides*), retrotransposons dynamics correlate with adaptation to microclimatic stress conditions, demonstrating that mobile element activity can serve as a marker of environmental response. In the allopolyploid *Spartina anglica*, retrotransposon mobilization following hybridization events contributed to genomic restructuring during speciation. The 160 Gbp fork fern (*Tmesipteris oblanceolata*) genome the largest known eukaryotic genome is dominated by repetitive elements, illustrating how transposon proliferation can drive extreme genome size variation in plants. In the rice blast fungus *Magnaporthe oryzae*, transposable element dynamics drive population divergence and host adaptation, with implications for understanding plant-pathogen coevolution. Diversity is an indicator of vital and healthy ecosystems (Rosenberg, 2024). Horizontal gene transfer occurs mainly, but not exclusively, in prokaryotes, plasmids, retroplasmids, phages, and transposons, which are conserved in eukaryotes or in highly developed organisms as mobile genetic elements of DNA and RNA (Van Regenmortel, 2020, 2018). Throughout the history of life, viruses and mobile genetic elements have interacted extensively with cellular organisms. Evidence suggests that viruses and mobile elements have contributed substantially to the evolution of fundamental cellular processes, including genetic recombination, aspects of transcription regulation, and transposition mechanisms in eukaryotic cells (Gozashti et al., 2025; Koonin et al., 2015; Koonin, 2016; Chuong et al., 2016; Krupovic and Koonin, 2015; Forterre and Prangishvili, 2009). Consequently, multiple viral infections or mobile elements are necessary for genome evolution (Kalendar et al., 2021; Kalendar and Karlov, 2023). These exogenous infections provide a basis for the complex mechanisms of genetic information observed today. They contribute to the development of cellular functions and adaptation of organisms to their environment (Kalendar et al., 2020; Sproul et al., 2023; Baumel et al., 2002). Thus, viruses play a key role in the evolution of life, acting not only as agents of infection but also as a driving force for genetic change (Legendre et al., 2014). Thus, intragenomic repetitive elements of viral or other origins are genetically decisive factors for evolutionary innovation and species diversity. The male Y chromosome provides an interesting example, being particularly poor in coding DNA and enriched in repetitive sequences, including elements of potential viral or transposon origin, although the precise evolutionary history of these elements remains an area of active research (Green et al., 2009; Petr et al., 2020). There is still a heated debate regarding the significance and function of non-coding DNA. Proponents of the junk DNA hypothesis, according to which most of our genetic material is wasted with no function, argue that some plants have a disproportionately larger number of genes than animals and humans (Palazzo and Gregory, 2014; Fernandez et al., 2024). However, it has been shown that most non-coding DNA is transcribed into RNA but is not converted into proteins. RNA transcripts perform high-level regulatory and control functions, the complexity of which is largely beyond our understanding (Portin, 2009; Van Regenmortel, 2004). However, many RNA elements are extremely short-lived and unstable, and their functions are not well understood. Similar to the central nervous system, when we discuss neural networks, the complex relationships in gene regulation can be described as genetic networks. A single gene rarely encodes a specific protein; however, the same nucleotide sequence can perform many different functions in different contexts. Many genes are transcription factors that initiate entire cascades of complex genetic regulation depending on the systemic context. In this respect, the state of the system is not determined by the state of its individual components; rather, the individual components follow the state of the system, which, in epistemology, represents a huge difference. The same applies to viruses and bacteria as well. Robert Koch’s discoveries in bacteriology in the second half of the 19th century were a scientific breakthrough when infectious diseases were dominated by medical thinking, but from today’s perspective, they nevertheless represent a one-sided reductionist approach. Koch grew individual bacterial strains as “pure cultures” in a nutrient solution and then transferred them to an organism (mice or guinea pigs), thus provoking disease. This hostile image of microbes persists. Bacterial and viral monocultures are rare in natural environments, where microorganisms typically exist within complex communities. Life forms have generally adapted to their environments as part of a microbial community. Isolating individual microorganisms from their primary environment changes their genotypes and phenotypes over time. In most cases, bacterial pathogenicity is attributed to viral elements (phages and plasmids). Cholera, diphtheria, botulism, dysentery, scarlet fever, and many other diseases are caused by bacterial exotoxins, which enter bacteria through phages (Brussow et al., 2004). Thus, the pathogenicity of microbes is determined not only genetically but also by the environment. Therefore, viruses and bacteria never live as separate entities but only in the context of an organism. Thus, intragenomic mobile elements and viruses are genetic elements capable of integrating into the host genome and influencing its evolution (Kalendar et al., 2000, 2020). They can serve as a source of genetic variability, promoting adaptation and the emergence of new functions. Their role extends beyond pathogenicity, highlighting the importance of viruses as driving forces in biological evolution.

## 2 Extended tandem arrays of retrotransposons: a challenge for long-read sequencing in plant genomes

Recent studies have identified multiple extended tandem arrays of retrotransposons within different plant species, including ferns (Kalendar et al., 2020). The formation of such extended tandem arrays appears to be a characteristic feature of plant genomes and represents one of the most challenging targets for accurate genomic characterization. These retrotransposon arrays, which can span tens to hundreds of kilobases, arise through successive rounds of retrotransposition, unequal crossing-over, and other recombination-based mechanisms that amplify repetitive sequences in head-to-tail orientation.

The biological significance of these extended tandem arrays extends beyond their role as genomic “passengers.” Retrotransposon arrays contribute to centromeric (Chang et al., 2019; Chabot et al., 2024), pericentromeric heterochromatin and telomeric organization (George et al., 2010), influence local recombination rates, serve as substrates for the generation of small interfering RNAs involved in epigenetic regulation, heterosis and can harbor regulatory elements that affect the expression of neighboring genes. In ferns, which possess some of the largest known eukaryotic genomes, extended retrotransposon arrays represent a particularly prominent genomic feature that has contributed significantly to genome size expansion. Understanding the structure, distribution, and evolutionary dynamics of these arrays is therefore essential for comprehensive genome annotation and functional interpretation.

However, the accurate sequencing and assembly of extended tandem arrays poses formidable technical challenges. Complex chromosomal regions containing long retrotransposons that themselves form extended tandem arrays require exceptionally high-quality, high-molecular-weight DNA for correct resolution. When individual retrotransposon units within an array span 5–15 kb and the entire array extends over 50–200 kb or more, only sequencing reads that exceed the length of individual repeat units can provide the phasing information necessary to distinguish between copies and accurately reconstruct the array structure.

Long-read sequencing technologies, particularly nanopore sequencing with its capability to produce ultra-long reads exceeding 100 kb, represent the sole feasible method for resolving these intricate repetitive structures. However, the realization of this potential is critically contingent upon the quality of the DNA. The production of ultra-long reads necessitates intact, high-molecular-weight DNA templates. Any fragmentation of the initial material, whether due to mechanical shearing during extraction, enzymatic degradation, or chemical damage from contaminants, directly constrains the maximum achievable read length and, consequently, the ability to span and accurately resolve genome sequences. For plant samples, which are especially susceptible to the presence of interfering secondary metabolites, attaining the DNA quality required for ultra-long-read sequencing of extended retrotransposon arrays constitutes a significant technical challenge. Moreover, even when long reads are successfully obtained, the presence of polysaccharides and other macromolecular contaminants can result in sequencing artifacts. These contaminants can cause pore blockages and current fluctuations, leading to systematic errors that are particularly pronounced in repetitive regions. This creates a compounding problem: the genomic regions most in need of long-read resolution are also those most susceptible to contamination-induced artifacts. Accurate characterization of extended repetitive regions in eukaryotic genomes is essential for comprehending plant genome evolution and function. This process necessitates not only long reads but also those derived from exceptionally pure, high-molecular-weight DNA preparations. This requirement directly informs the technical considerations discussed in the subsequent section.

## 3 Genome integrity as a bottleneck for long-read sequencing: macromolecular contaminants and the need for universal purification

The conceptual framework outlined above, which regards genomes as evolutionary archives of repetitive and mobile element activity, imposes specific and rigorous requirements on the technical aspects of genome analysis. Accurate interpretation of repetitive elements, structural variants (SVs), and mobile element insertions is critically dependent on the precise representation of long-range sequence information. This representation is feasible only when the initial DNA material retains its native high-molecular-weight structure. Fragmented or contaminated DNA preparations disproportionately compromise repeat-centric analyses in several ways. Firstly, extended repetitive regions are systematically underestimated when DNA fragmentation leads to assembly collapse, merging multiple repeat units into artificially shorter arrays. Secondly, SV detection becomes unreliable when DNA breaks occur within or near repeat sequences, resulting in erroneous calls of insertions, deletions, and inversions. Thirdly, retrotransposon length polymorphisms, which are crucial markers of genome evolution, cannot be accurately assessed when template integrity is compromised. Fourthly, epigenetic modifications within repetitive regions, including DNA methylation patterns that regulate transposon activity, are subject to artifacts when contaminating polysaccharides or phenolics interfere with native modification detection. For plant genomes, which present particular challenges due to high polysaccharide and secondary metabolite content, these technical considerations are especially critical. Thus, achieving the biological insights described in the previous section necessitates overcoming the sample preparation challenges outlined below.

The pinnacle of DNA and RNA-sequencing technology involves the analysis of individual molecules. The prospect of whole-genome long-read sequencing will most likely persist with nanopore technologies. It does not necessarily have to be in its current form, using an enzymatic complex, but in any other variant in which individual molecules of any nucleotide composition and length are analyzed as they pass through the pore and are analyzed nucleotide by nucleotide. Given the prospects of nanopore sequencing, there is a significant problem associated with the analysis of the native genomic DNA isolated during cell lysis. This problem is related to the presence of other macromolecules in the cell that are covalently and mechanically bound to high-molecular-weight nuclear DNA. Existing commercial or routine approaches to nucleic acid isolation generally do not allow for mechanical separation of polysaccharide macromolecules or other types of high-molecular-weight polymer molecules from nuclear DNA. The initial category comprises high-molecular-weight compounds, including polysaccharides and polyphenols, and low-molecular-weight substances such as humic substances. The presence of chemical or mechanical crosslinks between DNA chains, as well as contaminants interwoven with DNA, results in partial or complete inhibition of nanopore sequencing and the emergence of artifacts in the sequencing data. The quality of nucleic acids directly affects the artifacts encountered during nanopore sequencing. The inhibition of nanopore sequencing is associated with the mechanical intertwining of DNA with polysaccharides, which prevents the full advancement of DNA molecules through the pores during long-read sequencing. It is essential to eliminate all substances from the sample and use nucleic acids from the biological materials for further analysis. Isolation of DNA or RNA from biological materials is challenging because of the diversity and complex composition of the material itself. The biological materials include cells and tissues. Cells present in liquid media, such as blood, lymph, milk, urine, and feces, as well as those in culture, on agarose or polyacrylamide gels, in soil, or in solution, typically contain substantial amounts of contaminants. These contaminants must be eliminated from DNA or RNA before conducting molecular biological experiments. Many existing methods for isolating and purifying nucleic acids face significant limitations, often yielding suboptimal recovery rates and incomplete removal of contaminants. The presence of high molecular weight components that are mechanically linked to nucleic acids and proteins remains a persistent challenge.

The absence of polysaccharides in a mixture with nucleic acids is extremely important because their presence leads to the formation of a mechanical mixture that is often difficult to separate by conventional purification methods, including column technologies and the use of organic solvents. If the tissue being studied is rich in polysaccharides, DNA separation becomes particularly challenging because it remains mechanically bound to the polysaccharides. If there are active functional groups in the polysaccharides, DNA may form additional covalent bonds with them. When such mixtures are stored, the number of covalent bonds can increase, potentially leading to a loss in the ability of DNA to denature and reduced performance in nanopore sequencing.

All types of amplification, including PCR and isothermal methods, depend on effective unwinding and access to target sequences (Kalendar, 2025). Polysaccharides reduce the effective unwinding of long DNA fragments, which reduces the efficiency of amplification by inhibiting enzymes or physically blocking access to the DNA.

Electroelution represents a highly promising technique for the isolation and purification of nucleic acids and proteins from crude samples. This method effectively separates DNA from other compounds, including high-molecular-weight substances such as polysaccharides, polyphenols, pigments, and humic substances, which may interfere with subsequent DNA quantification and amplification. The method and electroelution apparatus are predicated on a straightforward yet effective principle: charged molecules, such as nucleic acids or proteins, moving in a constant electric current can be neutralized with a concentrated salt solution, thereby halting their movement entirely. This principle has been long established and has been employed for electroelution using a concentrated NaCl solution in the V-channels of electrophoresis systems (Zarzosa-Álvarez et al., 2010). Nucleic acids and proteins are completely arrested in front of a concentrated salt solution, resulting in their accumulation in the channel with electrophoresis buffer (Kalendar et al., 2024, 2023). This facilitates the isolation of various types of nucleic acids and proteins from a diverse array of biological sources. It has demonstrated particular efficacy for challenging samples, such as those derived from blood, soil, herbarium specimens, feces, and tissues rich in secondary metabolites, polysaccharides, and pigments. This approach offers a simple and versatile option for purification that warrants consideration as part of a comprehensive sample preparation strategy, particularly for samples where conventional methods fail to achieve adequate purity.

## 4 Key challenges and actions

To operationalize the conceptual framework delineated above, the plant genomics community should prioritize the following specific objectives:

### 4.1 Development of repeat-aware benchmarks

Current genome assembly and variant calling benchmarks inadequately evaluate performance in repetitive regions. The community should establish standardized reference datasets with validated repeat annotations, curated tandem repeat length polymorphisms, and characterized mobile element insertions across diverse plant taxa. These benchmarks should facilitate systematic comparison of assembly algorithms, variant callers, and annotation pipelines, specifically for their efficacy in handling challenging repetitive sequences.

### 4.2 Standards for long-read sample quality control

A community-endorsed minimal quality control panel should be established, specifying acceptable ranges for spectrophotometric ratios (A260/A280, A260/A230), fluorometric quantification protocols, fragment length distribution requirements, and functional validation metrics. Plant-specific thresholds should be developed to account for the unique challenges posed by polysaccharides, phenolics, and secondary metabolites.

### 4.3 Best-practice pipelines for repeat and structural variant analysis

Standardized, well-documented computational workflows should be developed and maintained for transposable element annotation, tandem repeat genotyping, and structural variant detection in plant genomes. These pipelines should be tested across taxonomically diverse species and made available through established bioinformatics repositories with clear versioning and reproducibility standards.

### 4.4 Community reference datasets

To enhance existing genomic resources, it is imperative for the scientific community to curate high-quality, long-read sequencing datasets from representative plant species. These should encompass genomes that present challenges due to high repeat content, polyploidy, and known difficulties in polysaccharide extraction. Furthermore, these datasets must be accompanied by corresponding quality control documentation, thereby enabling researchers to evaluate the correlation between sample quality metrics and subsequent analytical outcomes.

### 4.5 Limitations and counter-perspectives

It is essential to recognize the limitations inherent in the framework presented herein. The role of viruses and mobile genetic elements in fundamental cellular processes remains an area of active research, and alternative hypotheses concerning the evolution of transcription, translation, and recombination mechanisms warrant ongoing exploration. Not all repetitive DNA fulfills functional and structural roles, and the debate surrounding “junk DNA” reflects a legitimate scientific discourse regarding the proportion of genomes subject to selective pressure. Additionally, while electroelution and other physics-based purification techniques show potential, no single method can be universally optimal across all plant tissues and species. The recommendations provided should be considered as a foundation for community dialogue rather than as definitive solutions.

## 5 Conclusion

Modern molecular and genetic analyses have reached technological peak in terms of sequencing throughput, read length, and resolution. However, this progress has revealed a fundamental conceptual gap between data generation and biological understanding. The primary challenge is no longer the ability to sequence genomes but the ability to obtain biologically meaningful, reproducible, and interpretable information from complex genomic systems. Genomes are collections of protein-coding genes. They are dynamic systems shaped by repetitive and mobile genetic elements, viral integration, and long-term interactions with the environment. These components constitute a substantial fraction of genomic content and encode a historical record of evolutionary processes that cannot be captured by gene-centric or reductionist approaches alone. Ignoring this complexity leads to a systematic bias in genome interpretation, particularly in comparative genomics, population genetics, and evolutionary biology. Long-read sequencing technologies, especially nanopore-based approaches, provide unprecedented opportunities to analyze genome architecture at the native scale. However, their full potential is critically dependent on the integrity and purity of the high-molecular-weight nucleic acids. Contamination by polysaccharides and other macromolecules represents a fundamental bottleneck that cannot be resolved by incremental optimization of existing extraction protocols. Instead, a conceptual shift toward universal physics-based purification strategies that preserve the native DNA structure and eliminate inhibitory components at the molecular level is required. Electroelution-based purification offers a promising approach that has demonstrated efficacy for the isolation of intact nucleic acids from a wide variety of biological materials, while preserving their suitability for long-read sequencing, PCR-based genotyping, and genome-wide fingerprinting. More importantly, this exemplifies the broader principle that technological advances in genomics must be accompanied by equally rigorous advances in sample preparation, quality control, and system-level thinking. Therefore, the Grand Challenge for molecular genetics is not the development of yet another analytical pipeline but the integration of genome integrity, repeat biology, and system-level interpretation into a coherent experimental and conceptual framework. Only by treating the genome as an evolving, interconnected system, rather than as a static collection of genes, can we fully exploit modern sequencing technologies and move from descriptive genomics toward genuine biological understanding.
